# High levels of DEK autoantibodies may predict early flare following cessation of anti-TNF therapy in juvenile idiopathic arthritis

**DOI:** 10.1186/1546-0096-12-S1-P8

**Published:** 2014-09-17

**Authors:** Nirit Mor-Vaknin, Miguel Rivas, Maureen Legendre, Y Yuanfang, Anne Johnson, Bin Huang, Yuki Kimura, Lili Zhao, Steve Spalding, Paula Morris, Beth Gottlieb, Karen Onel, Judyann Olson, Barbara Edelheit, Michael Shishov, Larry Jung, Elaine Cassidy, Sampath Prahalad, Murray Passo, Timothy Beukelman, Jay Mehta, Kara Schmidt, Ed Giannini, Daniel Lovell, David Markovitz

**Affiliations:** 1 University of Michigan, Ann Arbor, USA; 2 Infectious Diseases, University of Michigan, Ann Arbor, USA; 3 Cincinnati Children's Hopstial Medical Center, Cincinnati, USA; 4 Joseph M. Sanzari Children’s Hospital, Hackensack, USA; 5 Cleveland Clinic, Cleveland, USA; 6 Arkansas Children’s Hospital, Little Rock, USA; 7 Cohen Children’s Medical Center, New York, USA; 8 Comer Children’s Hospital, Chicago, USA; 9 Children’s Hospital of Wisconsin, Milwaukee, USA; 10 Phoenix Children’s Hospital, Phoenix, USA; 11 Children’s National Medical Center, Washington DC, USA; 12 Children’s Hospital of Pittsburg, Pittsburg, USA; 13 Emory, Atlanta, USA; 14 Medical University of South Carolina, Charlestown, USA; 15 Children’s Hospital of Alabama, Birmingham, USA; 16 Children’s Hospital at Montefiore, Bronx, USA; 17 Kosair Children’s Hospital, Louisville, USA

## Introduction

The nuclear oncoprotein DEK is a biochemically distinct, pro-inflammatory protein that is a chemoattractant for neutrophils and T-cells. High levels of DEK autoantibodies have been found in several autoimmune diseases including juvenile idiopathic arthritis (JIA), but their role in disease pathogenesis is unclear.

## Objectives

Since DEK and DEK autoantibodies can contribute to the development of immune complexes and joint inflammation, we suggest that DEK antibody levels can predict disease flare with discontinuation of anti-TNF therapy.

## Methods

In 16 pediatric rheumatology centers, sera samples were collected from 137 children with polyarticular JIA on anti-TNF therapy. Therapy was stopped after 6 months for patients with clinically inactive disease (CID). Disease activity was then monitored for 14 months or until disease flare. DEK antibody levels were measured by ELISA in sera collected at time of enrollment, disease flare off therapy, or end of study. DEK antibody levels relative to healthy controls were calculated by area under the curve (AUC), expressed as unit-free ratios.

## Results

103 female and 34 male patients with polyarticular JIA were enrolled, mean age 11.3 years and disease duration of 5.0 years (77% were on etanercept, 18% adalimumab, 5% infliximab, and 40% concurrent methotrexate). 31 patients discontinued the study for various reasons, including loss of CID during therapy. 39 patients flared within 14 months of stopping therapy, but 67 subjects had no flare within those 14 months. In 89 patients’ samples collected at the end of the study or at time of flare, DEK antibody levels compared to healthy controls ranged from -0.69 (some patients had lower antibody levels than did healthy controls) to 0.83, mean difference of 0.068 (Q1-Q3 of -0.25-0.28 and 0.025 (SD, 0.39). High levels of DEK antibodies, mean and SD of 0.164 ± 0.39, with 95% confidence interval of (0.02, 0.31), were detected in 30 of the patients that flared within 14 months as compared to lower levels of DEK antibodies (-0.05 ± 0.39, 95% confidence interval of (-0.15, 0.05)) measured in 59 of the patients with no disease flare for 14 months (Student-T, P=0.016). Thus, patients that experience flare within 14 months of stopping anti-TNF therapy have significantly increased levels of DEK antibodies compared to patients that maintained their CID till the end of the study.

## Conclusion

In children with polyarticular JIA on anti-TNF therapy that maintain CID for at least 6 months while on therapy, high DEK antibody levels may correlate with flare within the first 14 months after stopping therapy. This study suggests that DEK antibody levels might predict the outcome of discontinuation of anti-TNF therapy.

## Disclosure of interest

N. Mor-Vaknin: None declared., M. Rivas: None declared., M. Legendre: None declared., Y. Yuanfang: None declared., A. Johnson: None declared., B. Huang: None declared., Y. Kimura: None declared., L. Zhao: None declared., S. Spalding: None declared., P. Morris: None declared., B. Gottlieb: None declared., K. Onel: None declared., J. Olson: None declared., B. Edelheit: None declared., M. Shishov Consultant for: Novartis, Amgen, L. Jung Consultant for: AbbVee, E. Cassidy: None declared., S. Prahalad: None declared., M. Passo: None declared., T. Beukelman Grant / Research Support from: Pfizer, Consultant for: Novartis, Genentech, UCB, McKesson Health Solutions, Crescendo, J. Mehta: None declared., K. Schmidt: None declared., E. Giannini: None declared., D. Lovell: None declared., D. Markovitz: None declared.

